# A Lightweight Method for Detecting and Correcting Errors in Low-Frequency Measurements for In-Orbit Demonstrators

**DOI:** 10.3390/s24041065

**Published:** 2024-02-06

**Authors:** María-Ángeles Cifredo-Chacón, José-María Guerrero-Rodríguez, Ignacio Mateos

**Affiliations:** Electronic and Electromagnetic Design Group, Escuela Superior de Ingeniería, University of Cádiz, Avda. de la Universidad, 10, E-11519 Puerto Real, Cádiz, Spain; josem.guerrero@uca.es (J.-M.G.-R.); ignacio.mateos@uca.es (I.M.)

**Keywords:** LEO orbit, proton, heavy ion, Single-Event Upset (SEU), Multiple-Bit Upset (MBU), flash memory reliability, satellite, electronic

## Abstract

In the pursuit of enhancing the technological maturity of innovative magnetic sensing techniques, opportunities presented by in-orbit platforms (IOD/IOV experiments) provide a means to evaluate their in-flight capabilities. The Magnetic Experiments for the Laser Interferometer Space Antenna (MELISA) represent a set of in-flight demonstrators designed to characterize the low-frequency noise performance of a magnetic measurement system within a challenging space environment. In Low Earth Orbit (LEO) satellites, electronic circuits are exposed to high levels of radiation coming from energetic particles trapped by the Earth’s magnetic field, solar flares, and galactic cosmic rays. A significant effect is the accidental bit-flipping in memory registers. This work presents an analysis of memory data redundancy resources using auxiliary second flash memory and exposes recovery options to retain critical data utilizing a duplicated data structure. A new and lightweight technique, CCM (Cross-Checking and Mirroring), is proposed to verify the proper performance of these techniques. Four alternative algorithms included in the original version of the MELISA software (Version v0.0) are presented. All the versions have been validated and evaluated according to various merit indicators. The evaluations showed similar performances for the proposed techniques, and they are valid for situations in which the flash memory suffers from more than one bit-flip. The overhead due to the introduction of additional instructions to the main code is negligible, even in the target experiment based on an 8-bit microcontroller.

## 1. Introduction

### 1.1. An Introduction to the Application Scenario

The Laser Interferometer Space Antenna (LISA) is the upcoming space-borne gravitational wave (GW) observatory led by the European Space Agency (ESA) in collaboration with NASA. For LISA, [1]. The magnetic environment from the interplanetary magnetic field and the spacecraft’s magnetic sources can contribute to non-gravitational forces that may disrupt GW detection. Consequently, to ensure the precise functionality of the observatory, it is imperative to employ low-noise, compact magnetometers capable of distinguishing the magnetic contribution from the overall measurements [2].

The Magnetic Experiments for the Laser Interferometer Space Antenna (MELISA) represent a series of compact magnetic measurement payloads able to discern interplanetary magnetic low-frequency drifts down to 100 µHz. MELISA serves as a technological demonstrator designed to characterize the low-frequency noise characteristics of a magnetic measurement system employing anisotropic magnetoresistive (AMR) sensors. To fulfill this objective, environmental magnetic field fluctuations during in-flight operations are mitigated using a cylindrical shield with concentric mu-metal layers encapsulating the triaxial AMR sensors [3,4].

To enhance the Technology Readiness Level (TRL) of precise magnetic sensing techniques for space applications, it is advantageous to conduct additional experiments utilizing in-orbit Demonstration and Validation (IOD/IOV) platforms. In this context, MELISA-II will be integrated onboard the UCAnFly CubeSat selected by ESA as part of the Fly Your Satellite program. Moreover, the European Union, within the framework of the H2020 IOD/IOV mission with the support of ESA, has chosen MELISA-III for inclusion in a 6U CubeSat called Σyndeo-2. The primary scientific objectives are to provide flight heritage to MELISA-II and MELISA-III, contributing to the advancement of magnetic sensing techniques for space-based GW observatories [5].

Scientific data obtained during MELISA in-flight operations are stored in non-volatile Flash Memory. To enable larger payload mass storage, highly scaled memory is required. Currently, Flash technology stands out as the most popular option to fulfill this role.

In space applications, it is well known that digital data in Low Earth Orbit (LEO) is sensitive to single-event effects (SEEs). These occur when highly energetic particles strike sensitive regions of an electronic component. In LEO, predominately two types of radiation induce SEE: galactic cosmic rays (GCRs) and protons trapped in Earth’s radiation belt. SEEs resulting in only one upset are called Single-Bit Upsets, or SBUs, while those resulting in several upsets are termed Multiple-Cell Upsets, or MCUs. SEE occurs when a particle changes the state of a circuit, causing one or more bit flips in memory cells or registers. If several upset cells belong to the same logic word, then the event is referred to as a Multiple-Bit Upset, or MBU.

Digital data corruption due to Single-event upset (SEU) events could occur during writing, reading, storage, transmission, or processing operations. The bits flipped introduce unintended changes to the original data. In addition, the presence of MBU is becoming more frequent with the evolution of the manufacturing process [6,7]. The errors in an MBU are normally caused by the same physical event and therefore affect memory bits that are close together, likely to affect two or more bits in the same word, causing an uncorrectable error. While SEUs remain a primary concern in space applications, the emergence of MBUs has become a matter to be addressed in the context of nanotechnology. The continuous die surface decreasing and the significant increase in bits/area density for new integrated devices (especially the new 3D flash memories) lead to an increase in MBU incidents for the same impact fluence (ions per area) [8]. Also, the work of Buddhanoy et al. [9] demonstrates significant degradation of the data retention characteristics of irradiated memories during long-term exposure.

### 1.2. Traditional EDAC Approaches

The payload’s scientific data integrity must be guaranteed before it is sent to Earth. If any data are potentially incorrect, at least it should be marked as ‘questionable’. It is preferable to have correct data over the entire data set since undetected incorrect data could lead to erroneous conclusions. To provide reliable scientific data efficiently, the implementation of error-control techniques is needed. These techniques are based on Error Detection and Correction (EDAC) codes.

For scientific data to be considered reliable, it should closely mirror the original data with minimal tolerance. Additionally, effective error correction must be processed in a short amount of time. It is necessary to find a balance between reliability and efficiency and, for each specific application case, to study what is more important to guarantee. This equilibrium must be found because prioritizing higher reliability can result in more computation time and/or greater memory usage, resulting in less efficiency.

According to the basics of Information Theory, an Error Correction Codes (ECC) encodes the original data, a *k*-bit input word defined as **u** = (*u*_0_, *u*_1_, *…*, *u_k–_*_1_), in an *n*-bit output word, word **b** [10]. The output word **b** = (*b*_0_, *b*_1_, *…*, *b_n–_*_1_) is a vector of *n* bits with (*n − k*) redundant bits appended; they are called parity or code bits. In a communication system, **b** is transmitted across a channel, which, in the case of noise, delivers the received word r = (*r*_0_, *r*_1_, *…*, *r_n–_*_1_). The error vector e = (*e*_0_, *e*_1_, *…*, *e_n–_*_1_) models the error induced by the noise in the channel in such a way that **r = b + e**. Error Correction Codes (ECCs) are also used to protect media storage devices.

Error correction codes can be implemented using hardware or software. Hardware is always an expensive solution, as it requires adding extra circuitry. Software solutions are not as expensive and allow the implementation of more complex coding schemes. Some codes and schemes are well-known and widely used, e.g., Triple modular redundancy (TMR). TMR is a hardware/software solution widely used in the onboard computer (OBC) present in aerospace systems such as satellites, spacecraft, and aircraft. Besides, the additional hardware required, another disadvantage of TMR is its large memory overhead [11]—three times the real capacity is required for redundancy. TMR can be employed to meet the reliability and safety requirements needed in critical systems. Most of the OBC in critical systems is based on redundant hardware, with high performance and high availability elements consisting of radiation-tolerant or Commercial Off-The-Shelf COTS components such as multicore 32-bit MCUs [12] or FPGAs [13,14].

Common ECCs employed to protect standard memories are Single Error Correction (SEC) or Single Error Correction-Double Error Detection (SECDED) codes [15]. SEC codes can correct an error in one single memory cell. SEC-DED codes can correct an error in one single memory cell as well as detect two-bit errors in two independent cells. In coding theory [16], the random error is the name given to one or more bits of error that are distributed randomly in the input word. Random errors can be one-bit or multiple. Single errors only affect a single memory cell, and they are provoked by SEU; however, as mentioned in Section 1.1, with the continuous increase in the integration scale, MBUs are becoming more frequent.

In space applications, more sophisticated codes are used [17,18]. For instance, the Hamming technique [19] is easy to implement in software or hardware. The limitation of the Hamming technique is its limited error correction abilities. Hamming code can correct single-bit errors and detect two-bit errors. The Matrix code [20] is a well-known code that combines Hamming codes with parity checks in a matrix and can correct up to two errors in each row, assuming that there is just one error in each column. Additionally, Column-Line-Code (CLC) [21] has been designed to tolerate MBUs in space applications. CLC is a modified Matrix code based on extended Hamming codes and parity checks. It uses extended Hamming codes and parity bits to correct up to two adjacent bits in error. The Parity per Byte and Duplication (PBD) [22] approach is a better approximation to the solution, but the drawback is the use of only one bit for checking parity. This is insufficient to keep meaningful information included in the scientific data.

The main problem with the above EDAC techniques is the redundancy required. Extra bits are used to detect and/or correct the possible errors that occurred, and redundant bits are added for each data word stored in memory. In this way, the amount of storage occupied for extra and redundant bits requires larger memories. An interesting work is presented in [23]. The authors provide a low-redundancy methodology called Flexible Unequal Error Control (FUEC), combining an algorithm and a tool. Then, they generate a series of ECCs [24] that greatly reduce the redundancy introduced while maintaining memory error coverage.

Also, the EDAC schemas imply overheads for the processor regarding power, program memory, data memory, and time. These overheads must be maintained as low as possible, especially in space applications.

### 1.3. Traditional EDAC Approaches for Cost-Saving and Power-Constrained MCU-Based Systems

Cost-saving measures are a common demand for CubeSat instruments. In nanosatellites, opting for Commercial-Off-The-Shelf (COTS) components instead of expensive radiation-hardened devices is a practical choice. As part of a cost-saving, constrained system, the MELISA instrument is based on a COTS 8-bit MCU. The choice of an 8-bit MCU is suitable for controlling a simple measuring process and storing data readouts, aligning with the power restrictions of a nanosatellite powered by solar panels. The energy-efficient architecture of an 8-bit MCU makes it a practical solution for this application.

The energy-efficient architecture of an 8-bit MCU makes it a practical solution for this application. Low-power consumption components are required to ensure proper operation, even with low energy generation captured from the satellite’s solar panels. Therefore, the selection of an 8-bit microprocessor is dictated by technical considerations, despite the potential drawbacks when executing complex code or at high-performance rates.

On the other hand, the MELISA main function is managing an electronic circuit controlling a magnetic sensor, taking some measurements, and saving them in memory with a critical and specific frequency. Consequently, whatever additional feature must be added is fixed to the established time.

The SEU and MBU errors to which the records stored in the Flash memories will be exposed have already been documented in the introduction of the article. Nevertheless, it is worth explaining the cause of the extended exposure to these risks. The experiment (providing the information) and other experiments are hosted on a nanosatellite (in the Low Earth Orbit). All of them are remotely controlled by Earth, which also manages the functioning of the nanosatellite itself. Considering this, communication with Earth only occurs when the position of the nanosatellite in orbit permits it, and this is a brief time window. Additionally, when that moment arrives, it must be shared among various communication tasks.

However, redundancy is not a critical resource in this scenario, as MELISA is equipped with 1 GB of Flash memory dedicated to information storage. The data generated during the experiment occupies less than half of the memory; consequently, the remaining half may be reserved for a simple data backup or any fault tolerance technique such as RAID 1 (data mirroring).

Implementing Hamming, Matrix Code, or Colum Line Code would require significant computational algorithms, CPU resources, program memory, and power consumption. Bearing in mind the features of the chosen MCU, these arguments discard the implementation of traditional EDAC codes in systems such as MELISA.

MELISA produces a data set composed of 10 bytes to code five types of information. Consequently, relying solely on 1 or 2-bit correction techniques is not enough to maintain data coherence. Neither of the above approaches can detect more than two error bits. Additionally, while the PBD approach is focused on byte-to-byte processing, it is only valid in cases where an odd number of errors per byte occur.

Other techniques capable of detecting and correcting more than 1 or 2-bit errors, such as linear block codes with exhaustive error-correcting capabilities (e.g., LDPC, Reed-Solomon Code), are not appropriate in applications such as MELISA. This is due to their potential to increase software complexity. While many of the algorithms underlying classical EDAC methods can be implemented effectively [25], it is not the most suitable choice due to the increased demand for memory and energy on this type of microcontroller, nor would it be suitable for the execution time.

This paper proposes a method to maintain information integrity called the Cross-checking and mirroring technique (CCM). It is positioned as an enhancement to the technique presented in the referenced paper [22] and is based on a combination of redundancy and error detection code. In contrast to classical EDAC, which tries to reduce redundancy, CCM employs 100% redundancy, leveraging the RAID 1 architecture combined with Cyclic Redundancy Check (CRC) as an error detection code. RAID 1, commonly known as mirroring, involves maintaining an exact duplicate (mirror) of the original data, offering fault tolerance by creating a redundant copy. CRC, a widely used error detection code, involves the calculation of a short, fixed-size checksum to detect errors in data storage and transmission.

CCM is implemented in software (bare metal programming) on an 8-bit microcontroller operating at 8 MHz, and the primary goal is to guarantee the integrity of information stored over an extended period. It is worth noticing that the proposed method focuses on maintaining the integrity of the data before transmission to Earth. It is not designed to guarantee the correctness of communication between the nanosatellite and Earth. A key focus is on achieving a small execution time and reduced power consumption, given the specific constraints of the MELISA application.

The significance of the CCM approach lies in its ability to address the dual vulnerability of both the original and mirrored memory copies to MBUs. By integrating RAID 1 and CRC, our method seeks to provide a robust solution for the preservation of information integrity, even in the event of simultaneous errors in both copies.

Traditional approaches often involve the computation of matrices or complex polynomials to obtain redundancy bits, which can be computationally expensive and require a long execution time. Our method aims to circumvent this limitation, ensuring the demanding execution time of the system. Through empirical validation, this paper aims to establish the efficacy of the CCM method in mitigating MBU-induced errors and safeguarding the integrity of information stored in space applications.

Some variations of the CCM scheme are compared before determining the definitive technique. Each variant uses different processes, strategies, and steps for checking the integrity of the data. All the presented versions aim to transmit to Earth as much scientific data as possible with less penalty in parameters such as power consumption, elapsed time, and storage requirements.

The rest of this paper is organized as follows: In Section 2, we present four alternative algorithms to restore the correct data and the merit indicators used to compare them. Section 3 details the results achieved for the four algorithms based on a real application. Section 4 analyzes and discusses the values obtained regarding the merit indicators, comparing the performance of the proposed techniques against others. Finally, Section 5 concludes this paper.

## 2. Algorithms: Definition and Test Procedure

The initial premise of this work assumes that the bit-flipping issues are primarily induced in flash memory due to the sensibility of the memory arrays to high-energy particle impacts. Failures in the microcontroller and other hardware components for communication (such as the I2C bus) are dismissed, considering that they present the corresponding Radiation Tolerance qualification.

The example mission experiment, MELISA, requires periodically storing a data set in the Flash memory. It is required to successively store a fixed block size of 12 bytes, which corresponds to 10 bytes of instrument readings accompanied by two CRC16 control bytes.

### 2.1. Analysis of Bit-Flippling Cases and Methods of Implementation

#### 2.1.1. The Cross-Checking and Mirroring Technique (CCM)

**Figure 1:**
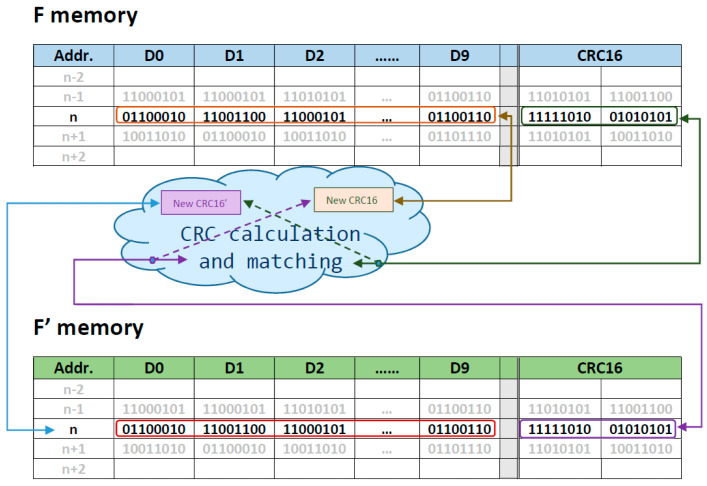
Graphical representation of the CCM technique.

The CCM technique is an enhanced version of the BPD scheme proposed in [22], trying to leverage its key features by combining data redundancy with an error detection code (Figure 1). In this context, in order to achieve greater robustness, the CRC16 is the chosen EDAC. In comparison to [22], this new approach avoids errors due to the parity bit method, especially in instances involving an even number of bit flips. The CRC method exhibits numerous advantages over the parity check. Specifically, CRC error correction allows detection of single-bit errors, double-bit errors, or errors occurring in adjacent bits. A parity bit detects only single-bit errors.

Next, four potential versions of the technique will be elaborated upon, delving into their details and evaluating the usefulness and convenience of each. Since all of them rely on data redundancy, two different storage areas are needed. Therefore, the flash memory is divided into two equal blocks named F and F’.

In order to identify bit errors, particularly bit-flipping, within the stored scientific data, an error detection code is introduced. The selected code is the Cyclic Redundancy Check Error of 2 bytes (CRC16). CRC is a widely employed method in digital networks and storage devices for detecting alterations in data. CRC16 involves binary division of the data bits by a predetermined divisor. The divisor is generated using polynomials. All the proposed versions require calculating the CRC16 for each scientific data point.

The writing process for the versions evaluated here is based on a Redundant Array of Independent Disks type one (RAID 1) for data storage in an array of hard disks, referred to as disk mirroring, which replicates data to more than one storage area. In this paper, F is the name used for the primary memory and F’ for the redundancy memory. However, unlike RAID 1, the payload MCU cannot write the data simultaneously in both flash memory areas. Instead, two sequential writing actions are executed.

The scientific datum, *data*(*i*), annexed to its corresponding CRC16 is called **DATA_WR**(i)and written in F. Its counterpart *data’*(*i*), along with the CRC, is written in F’ and called **DATA_WR’**(i).
Technique CCM: Write process    1.   The CRC is calculated for data(i) → **CRC_WR**(i)     2.   A new register is composed joining data(i) and CRC16 → **DATA_WR**(i)     3.   **DATA_WR**(i) is written twice, in F and in F’→ **DATA_WR**(i) and **DATA_WR’**(i)

The four proposed methods (#1, #2, #3, and #4) use the same writing process; however, they implement different reading processes, which are described below. It is important to note that, by the time the stored scientific data are read, it could have suffered from MBU, potentially resulting in a deviation from the original data.

In methods #1 and #3, the initial step in the reading process is a comparison between the *j-bytes* of *data*(*i*), read in memory F, and its counterpart *data’*(*i*), read in memory F’. If they are equal, the process concludes that the datum, *data*(*i*), is correct and therefore can be sent or used. Otherwise, some additional steps are undertaken to determine what the correct datum is, whether *data*(*i*) or *data’*(*i*). The method to find out this involves calculating the CRC for both and comparing each result to the CRC stored in F and F’. The valid datum is that whose CRC calculated matches the stored CRC.
Technique CCM (#1): Read process1.  A datum is read from F and F’ memories →         **DATA_RD**(i) = *data*(*i*) + **CRC_WR**(i)         **DATA_RD’**(i)= *data’*(*i*) + **CRC_WR’**(i) 2.  if (*data*(*i*) == *data’*(*i*)) then *data*(*i*) is correct     else     2.1. The CRC of *data*(*i*) in **DATA_RD**(i) is calculated → **CRC_RD**(i)     2.2. if (**CRC_RD**(i)== **CRC_WR**(i))              *data*(*i*) is correct and consequently *data’*(*i*) is corrupt         else         2.2.1. The CRC of *data’*(*i*) in **DATA_RD’**(i) is calculated→ **CRC_RD’**(i)         2.2.2. if (**CRC_RD’**(i)== **CRC_WR’**(i))                   *data’*(*i*)is correct and consequently *data’*(*i*) is corrupt                else                  *data*(*i*)and *data’*(*i*)are discarded 

In CCM (#1), the reading process relies on a *j-byte* comparison to determine if any byte is corrupted. CCM (#2) attempts to eliminate this stage, preventing an extensive comparison even if the *data*(*i*) were correct. In such instances, access to the backup flash memory would be unnecessary. Consequently, CCM (#2) initiates the verification process by checking if *data*(*i*) is correct by employing the CRC16. It is assumed that the majority of the data are correct; thus, this approach would decrease the average execution time and reduce power consumption.
Technique CCM (#2): Read process 1.   A datum is read from F memory → **DATA_RD**(i) = *data*(*i*) + **CRC_WR**(i) 2.   The CRC of *data*(*i*) in **DATA_RD**(i)is calculated→ **CRC_RD**(i) 3.   if (**CRC_RD**(i)== **CRC_WR**(i))          *data*(*i*) is correct      else        3.1. Read *data’*(*i*) in F’ → **DATA_RD’**(i)= *data’*(*i*) + **CRC_WR’**(i)        3.2. if (**CRC_RD**(i)== **CRC_WR’**(i))                *data*(*i*) is sent because **CRC_WR**(i) is corrupt             else                3.2.1. Calculate CRC for *data’*(*i*) → **CRC_RD’**(i)                3.2.2. if (**CRC_RD’**(i)== **CRC_WR’**(i))                          *data’*(*i*) is sent and consequently *data*(*i*) is corrupt                       else                         *data*(*i*) and *data’*(*i*) are discarded

CCM (#3) operates similarly to CCM (#1) but with the aim of restoring more data among the corrupted ones. It is intended for recovering data where the CRC field exhibits bit-flipping. To achieve this, a cross-comparison occurs between the CRC calculated from the read data in memory F and the CRC written in the F’ memory area.
Technique CCM (#3): Read process1. Required datum is read from F and F’        **DATA_RD**(i) = *data*(*i*) + **CRC_WR**(i)        **DATA_RD’**(i)= *data’*(*i*) + **CRC_WR’**(i)
 
2. if (*data*(*i*) == *data’*(*i*))
           *data*(*i*) is correct and sent
   else
   2.1.  The CRC is calculated for *data*(*i*) in **DATA_RD**(i) → **CRC_RD**(i)
   2.2.  if (**CRC_RD**(i)== **CRC_WR**(i))
            *data*(*i*) is correct and consequently *data’*(*i*) is corrupt
         else
         2.2.1. The CRC is calculated for *data’*(*i*) → **CRC_RD’**(i)
         2.2.2. if (**CRC_RD’**(i)== **CRC_WR’**(i))
                      *data’*(*i*) is correct and consequently *data*(*i*) is corrupt
                   else 
                      2.2.2.1 If (**CRC_RD’**(i)== **CRC_WR**(i))
                                *data’*(*i*) is correct and *data*(*i*) is corrupt
                              else
                                *data*(*i*) and *data’*(*i*) are discarded


The CCM (#4) is derived from the CCM (#2); however, similar to the CCM (#3), it incorporates additional steps in an attempt to recover more data among those that are incorrect. Once more, considering that the corrupted field may involve the CRC, a cross-comparison is conducted.
Technique CCM (#4): Read process 1. A datum is read from F → **DATA_RD**(i) = *data*(*i*) + **CRC_WR**(i)  2. The CRC of *data*(*i*) is calculated→ **CRC_RD**(i)  3. if (**CRC_RD**(i)== **CRC_WR**(i))         *data*(*i*) is correct     else        3.1. Read *data’*(*i*) in F’ → **DATA_RD’**(i) *data’*(*i*) + **CRC_WR’**(i)        3.2. if (**CRC_RD**(i)== **CRC_WR’**(i))                *data*(*i*) is sent and consequently **CRC_WR**(i) *is* corrupt             else                3.2.1. Calculate CRC for *data’*(*i*) → **CRC_RD’**(i)                3.2.2. if (**CRC_RD’**(i)== **CRC_WR’**(i))                          *data’*(*i*) is sent and                                 consequently, **DATA**(**i**) OR **CRC_WR**(i) *are corrupt*                       else                    3.2.2.1. if (**CRC_RD’**(i)== **CRC_WR**(i))                          *data’*(*i*) is sent and                                 consequently, **DATA**(**i**) OR **CRC_WR’**(i) *are corrupt*                        else                            *data*(*i*) and *data’*(*i*) are discarded

Various CCM techniques are analyzed to evaluate their efficiency based on the indicators outlined in Section 2.1.2.

#### 2.1.2. Evaluation Indicators

The CCM technique detects and recovers flipped bits in a data set stored in flash memory. To evaluate all versions of the technique, some qualitative and quantitative indicators are proposed. While the applicability of the technique extends to any system with flash memory, this paper focuses on its integration into the main program of an 8-bit MCU in a nanosatellite payload. Therefore, the indicators are tailored to this specific work environment, with a particular emphasis on the MELISA experiment. Subsequently, a detailed description of each indicator is provided.

- Energy consumption index (I): This index refers to the average energy required by the payload for both writing and reading actions, measured from the average current during the execution of the evaluated routine. In this test phase, only the microcontroller, memory, and minimum auxiliary digital circuits are in the on-state. Energy efficiency is a tight constraint in onboard electronics due to the limited power generation and storage capacity of nanosatellites.
- Writing time in the flash memory (***t_WR_***): The MELISA payload must perform the measurements within a precise time period. After each new measurement, the scientific data must be written before a new one starts. This constraint limits the duration of the writing process in the external flash memory.
- Reading time in the flash memory (***t_RD_***): in contrast to ***t_WR_***, the reading time is less constrained since the scientific data is required from the OBC in a 12-byte package one-to-one, and therefore the time can be tuned. However, since the OBC is required to read data from all the payloads and subsystems of the nanosatellite, it is essential to minimize this processing time.
- Discarded data number (**Ddn**): every version of the CCM technique aims to reduce the number of discarded data due to flipped bits. A lower **Ddn** value indicates a more effective technique.
- MCU Program Memory occupancy (**PMo**): The different techniques explore different strategies to mitigate the consequences of the corrupted data. To achieve this goal, they introduce additional instructions in the payload main program; as a result, the percentage of the MCU program memory is increased.
- MCU Data Memory occupancy (**DMo**): According to the actions described in the previous indicator, the MCU data memory are also increased by the additional instructions.
- Data Flash Memory occupancy (**FMo**): Modern designs tend to use SSD flash memory as scientific data storage. The MELISA payload incorporates a flash memory to fulfill the required storage capacity of the experiment. Redundancy involves storing multiple copies of data on the device to ensure valid data will be valid. Two copies of information and some EDAC help to ensure redundancy and correctness in space applications. The flash memory is faster than the older magnetic media but is over 12 times more expensive. This indicator estimates the increase in the price of the payload based on the chosen technique.

### 2.2. Methods

Each CCM technique is included in one branch of the main program for verification and assessment of the indicators. The Melisa payload is controlled through an OBC emulator, which serves as the tool for commanding the writing and reading operations of the external memory of the payload.

The CCM method is tested in a laboratory scenario with the MELISA payload of the device under test (DUT). The scientific data set is a synthetic one previously written in the Flash memory of MELISA. The synthetic data set contains all the possible faults that can be expected. More details of the data generation can be found in the next sub-section.

#### Indicators Assessment

The assessment of each indicator requires different procedures. They can be as simple as reading information in the integrated development environment (IDE) employed to develop the main program. The indicators achieved in this way are (a) the Program memory occupancy (*PMo*) and (b) the Data memory occupancy (*DMo*).

The method to determine the Data Flash memory occupancy (*FMo*) is related to the executed experiment; every type of activity is going to determine how much data are produced and stored. Melisa is intended to last 72 h, storing a new scientific datum every 200 ms. As a result, 1,296,000 write operations occur in the memory. Then, the memory occupancy is obtained by multiplying the number of writings by the number of bytes in one scientific datum. Since the Melisa scientific data block consists of 12 bytes, a storage requirement of approximately 29 bytes results.

However, for assessing the remaining indicators, more complex actions are undertaken. These are related to time, energy, and the discarded data rate, which are described below.

To assess the Discarded data number (*Ddn*) indicator, an analysis of the possible scenarios in the data upon MBU occurrences is required. Failures in data can be different depending on which bit is flipped in the scientific data. Additionally, it must be taken into account that both the primary and redundant memory, F and F’, can be affected. Table 1 defines the types of failures that can be found in the scientific data set, including the fault-free datum.

Based on Table 1, a synthetic benchmark is generated consisting of five data points for each type of error defined. With 16 types of faults (absence of error is also a type), the benchmark is composed of 80 data points in total. The idea is to define a fault-free data set and to inject faults according to those described in Table 1. Then, the benchmark is written in the MELISA flash memory. Following this, the benchmark is read four times, each time using a different version of CCM. The *Ddn* indicator is assessed to verify the capability to detect and correct the errors in each CCM version.

Time and energy consumption indicators are evaluated by means of the same benchmark written in the flash memory. The detailed process for each is as follows: For time-related indicators, extra instructions are added to the Melisa main program. The extra instructions are for driving an unused output port in the MCU in such a way that when the writing/reading period starts, the signal goes high and returns to low when it stops. An oscilloscope is then used to show the duration of the pulse.

- Writing time in flash (***t_WR_***) is a particularly critical parameter due to the tight timing required in the Melisa experiment. Roughly a million measurements take place during a MELISA experiment, and each must be written to the memory within a time of 10 ms. The averaging function of an oscilloscope is used for the multiple data acquired during the Melisa experiment, yielding the mean value of the writing time. It must be taken into account that the writing time should be the same for all versions, as the writing process is the same in each of them.
- The same method is applied to measure the *Reading time in the flash memory* (***t_RD_***). However, unlike the writing time, in the MELISA experiment, the reading process is commanded one by one. On the other hand, in each version of the CCM technique, the reading time varies depending on the type of error found in the data. This situation forces the manual measurement of the time for each readout. To achieve this, the benchmark stated before is written in the flash memory. Then, every time a new reading is commanded, the oscilloscope shows the reading time for one datum, and it is recorded in a spreadsheet. As the benchmark includes a total of eighty data points, the reading time for each technique version is obtained by calculating their average.
- Energy consumption index (I): a precision ammeter (Keysight 344,70 A, 7 ½ Digits) is used to measure the current demanded by the Melisa board while it is reading all the data in the benchmark in a row. The instrument is configured to measure current in the range of mA, as the maximum current drained by Melisa when in an idle state is 5.47 mA. It was also configured to optimize measurements by adjusting the smoothing level, response speed, and sampling interval.

## 3. Results

Firstly, each version of the CCM technique was executed on the 8-bit MCU of the Melisa board. Subsequently, the experiment was initiated to measure and write an extensive set of real data in the flash memory as scientific data. As the Melisa board is tested in the laboratory, there are no data errors in this collection of scientific data.

Afterward, in this fault-free scenario, the four versions of the CCM technique were validated, and all of them performed as expected. Anyway, it was checked that the introduction of the CCM scheme did not provoke delays that could affect the time constraints of the Melisa system.

The merit indicators were measured using the methods proposed. Table 2 shows the results in this first scenario, and they are compared to the results achieved with the original Melisa program, namely, without any CCM technique implemented.

In the second step, the synthetic benchmark emulating data errors caused by SEU and MBU is registered in the storage memory utilizing a program developed expressly for this purpose. The original program and the four different versions of the CCM scheme are then tested in the MELISA payload, and the indicators are obtained following the established method (see Table 3).

Note that the benchmark contains an equal number of data points for each error type (see Table 1), and the indicator ***t_RD_*** is the average of the time it takes to read each—the time depends on the position of the bit-flipped. For that reason, the best and worst cases are also included. It is worthwhile to notice that the best case for reading time could also be taken into account as another merit indicator, as it is supposed that most of the data are going to be correct.

It is a valuable point to detail the results obtained for the indicator Ddn. Table 4 gathers the number of errors that could be corrected and the number of errors that were not corrected with each version of CCM. The results are also expressed as a percentage of the relationship between the number of errors corrected/not corrected and the 16 types of failures. Notably, CCM v.3 results as the winning version according to this indicator.

## 4. Discussion

Various implementations of the CCM technique can be applied for error detection and correction in memories. Specific merit indicators have been proposed to apply the CCM technique in the context of the MELISA experiment. All the proposed CCM techniques have been tested on the mentioned payload. This evaluation aims to determine the most recommended technique for the purposes of MELISA and to detect any potential interference with the main tasks.

The obtained results for the indicators are depicted in Table 2, Table 3 and Table 4, each one for a different data benchmark. In the analysis of Table 2, it is worth noting some ideas about the indicators:

- The ***t_WR_*** (Write time) indicator confirms that the writing time constraint is fulfilled for Melisa. It is within the 10 ms required, despite the additional tasks included in the CCM approach. This is to calculate a CCR16, aggregate it into the scientific register, and write the whole register twice in the flash memory.
- The ***t_RD_*** (Read time average) indicator represents the average time for reading all the data in the first benchmark. Although there are no errors, the read time has increased due to the process of error detection by calculating the CRC16. CCM #1 and #3 present the slowest read time, whereas #2 and #4 are roughly the same as the original time.
- The memory indicators allow us to know how many bytes occupy the program after including the CCM technique. They represent the bytes occupied in the MCU program and data memory. As can be seen in Table 2, the extra amount of bytes in the program memory is approximately 10%, and in the data memory, it is 0%. Both results are acceptable for the chosen 8-bit MCU, which features 128 kB of program memory and 4 kB of data.
- Energy consumption, Deleted data numbers, and Robustness indicators are not discussed because they do make sense in a fault-free scenario. They will be reviewed based on the results obtained with the benchmark, including faults.

Table 3 shows the results after the test with the synthetic benchmark containing faults. It is worth remembering, in this case, that most of the data are incorrect, and therefore the CCM method tries to find out if the datum in the primary or redundancy memory is correct or, otherwise, fill the readout with 0 to warn that it is not a valid datum.

- As can be expected, the ***t_WR_*** indicator for the synthetic benchmark remains equal to that in Table 2.
- The ***t_RD_*** indicator, for all types of data, is expected to be longer than in the previous test. Once a datum has been read from the primary memory and an error is detected, the CCM method must continue reading the redundant memory, trying to find the correct data. As can be seen in Table 3, once again, CCM #1 and #3 present the slowest average read time, whereas CCM #2 and #4 are faster.
- The best-case value provides additional information about the read time in the event that the datum is correct. The worst-case scenario shows the read time employed by the CCM technique when it detects an error and tries to find out what the correct scientific data are, making use of the redundancy memory. The best-case value is especially important because it is supposed that most of the data are going to be correct, and therefore it could be closer to real-time than the average evaluated value.
- The memory indicators change regarding the first benchmark.
- Energy consumption: The MELISA payload operates under tight consumption constraints because there are many other experiments hosted on the nanosatellite, all of which must share the power supply. However, as can be noticed from Table 3, the increase in consumption resulting from the introduction of the CCM scheme can be neglected, even in the worst-case scenario when the program spends more time reading the correct data. The maximum increase in consumption is less than 110 µA.
- Discarded data number: CCM #3 presents only 6 discarded data out of 16, namely 37.5%. Table 1 depicts the 16 types of error that can occur depending on the position of the bit suffering from bit-flip, whether it is in the CRC or the data fields, both in a primary memory datum and a redundancy memory datum. This means that CCM #3 is capable of recovering more incorrect data than any other.
- A robustness indicator is included to show if the algorithm could send incorrect data in the unlikely case of the bit flipped being in the same position of the datum, either in the primary or the redundancy memory. CCM #2 and #4 ensure this does not occur, unlike #1 and #3, which are not able to detect this type of error.

As a conclusion, for the MELISA experiment, the most interesting CCM technique is that which presents a lesser number of discarded data, CCM #3.

A quantitative evaluation of the CCM error detection/correction capacity is not feasible as conducted with classical EDAC methods; nevertheless, Table 1 and Table 4 could be considered as an assessment of the performance of the proposed technique. The data reading in Melisa is not a strict constraint; however, it is very important to guarantee the data integrity before sending it to earth. If there are data points that may be incorrect, at least they should be marked as ‘questionable’.

Even in the case of requiring less time for reading data, the CCM technique is still valid after some modifications. An analysis of the Melisa scientific data shows that only some bytes in the data are important and cannot be corrected on earth. In consequence, it is possible to apply the CCM technique only to those bytes. In that way, the reading time can be reduced. This modification can be extrapolated to other experiments in which scientific data present similar conditions.

## 5. Conclusions

In this paper, we have explored and proposed a novel method for mitigating Multiple Bit Upsets (MBUs) in the storage memory of systems operating in space. The proposed scheme is called Cross-checking and mirroring (CCM). The approach uses Cyclic Redundancy Check (CRC) as an error detection code, calculated for each data word. It is then appended to the word data before being written to the memory and in a backup memory, following the principles of the RAID 1 architecture. The approach demonstrates promising potential for ensuring the sustained integrity of information stored in a challenging space environment.

Four techniques are proposed, presenting differences in the method applied to determine the correct datum in a reading process. Along with the technique, some merit indicators have been provided to evaluate which of them has the best characteristics.

To validate the CCM technique, it was tested on an in-orbit demonstrator called Melisa, which consists of an 8-bit MCU-based payload hosted in a nanosatellite. MELISA takes and stores a large number of measurements to characterize a magnetic sensor. The MELISA data word is composed of ten bytes, each representing different information acquired during the experiment.

This validation process was based on two case studies. First, the four CCM techniques were tested by using a fault-free set of real scientific data. In the second case study, a synthetic benchmark containing errors was used. The obtained results demonstrated the effectiveness of the proposed approach to detect and correct errors in memory systems. Unlike a technique based on byte-parity, the CCM technique is valid, although more than one bit is flipped due to SEU or MBU. Moreover, the evaluated indicators allowed us to identify the CCM technique with the best features.

All the versions have been validated and evaluated according to certain merit indicators. The choice of using one CCM technique over another depends on the application and the required performance in each case.

In our target experiment, MELISA, the CCM technique #3 achieved the best selection of dataset (there are two sets available thanks to the redundancy) to be transmitted to Earth. Our investigation focused on the recognition that both the original and mirrored memory copies can be susceptible to MBUs. By integrating RAID 1 and CRC, the proposed method aims to address the dual vulnerability of both copies, offering a comprehensive solution to MBU-induced errors.

The results indicate that the proposed method successfully detects and corrects MBU-induced errors, ensuring the sustained accuracy and reliability of the stored information over time. In addition, unlike the PBD approach, which is only valid in cases where an odd number of errors per byte occurs, CCM works successfully for both even and odd errors. The integration of CRC complements the redundancy aspect by providing an additional layer of error detection without imposing a substantial computational burden.

In conclusion, the presented research contributes a viable and adaptive solution to the persistent challenge of MBU-induced errors in memory systems working in space. By combining the principles of RAID 1 and CRC, our proposed method offers a reliable means to safeguard data integrity, making strides toward enhancing the resilience of electronic systems operating in the demanding conditions of space exploration.

## Figures and Tables

**Table 1:** The 16 types of possible faults are considered for evaluating the CCM method. The symbol ‘
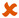
’ highlights one or more flipped bits in that data field, while the ‘
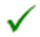
’ symbol indicates a data field without faults.

Primary Memory (F)			Redundant Memory (F’)	
DATA_WR(i)			DATA_WR(i)	
Data(i)	CRC_WR		Data’(i)	CRC_WR’
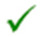	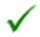		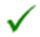	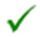
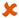	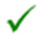		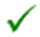	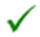
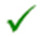	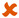		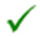	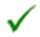
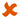	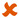		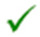	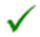
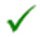	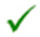		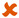	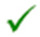
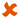	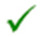		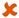	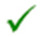
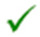	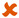		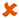	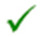
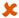	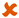		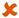	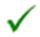
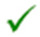	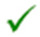		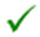	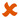
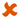	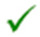		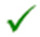	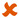
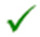	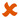		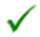	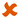
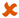	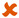		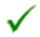	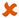
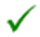	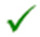		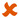	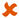
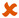	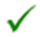		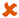	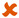
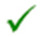	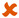		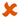	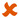
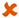	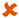		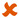	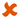

**Table 2:** Indicators obtained by using a fault-free data set.

Indicator	Original	CCM v.1	CCM v.2	CCM v.3	CCM v.4
**t_RD_**—Read time average (us)	260.6	300.2	264.5	300.2	264.3
Read time (Best case) (us)		300.1	254.9	300.1	254.9
Read time (Worst case) (us)		300.2	271.7	300.3	271.7
**t_WR_**—Write time (us)	151	662.7			
**Ddn**—Discarded data	It is not necessary, 0 discarding happens.				
Robustness	It is not necessary, no error is possible.				
**PMo**—Program memory (bytes) (*)	5048	5510	5638	5720	5740
**DMo**—Data memory (bytes) (*)	431	431			
**FMo**—Flash occupancy (Mbytes)	12.36	29.66			
**I**—Energy consumption Index (mA)		Not measured			

* The program and data memory occupancy are related to the complete program for managing the MELISA payload.

**Table 3:** Indicators obtained by using the benchmark with flipped bits included.

Indicator	Original	CCM v.1	CCM v.2	CCM v.3	CCM v.4
**t_RD_**—Read time average (us)	260.6	458.26	442.28	457.66	442.85
Read time (Best case) (us)		300.2	267.3	300.2	267.3
Read time (Worst case) (us)		555.7	528.7	566.8	547
**t_WR_**—Write time (us)	151	662.7			
**Ddn**—Discarded data	0	8	8	6	7
	0%	50%	50%	37.5%	43.75%
Robustness (*)	No	No	Yes	No	Yes
**PMo**—Program memory (bytes) (**)	5134	5510	5638	5720	5740
**Memory increasing** (**%**)	-	+7.3%	+9.82%	+11.4%	+11.8%
**DMo**—Data memory (bytes) (**)	431				
**FMo**—Flash occupancy (Mbytes)	12.36	29.66			
**I**—Energy consumption Index (mA)	7041	7065 +0.34%	70,955 +0.79%	7075 +0.48%	7104 +0.895%

* By using CCM techniques, no data are sent when data(i) and data’(i) have errors, even though the errors match. ** The program and data memory occupancy are related to the complete program of the MELISA payload.

**Table 4:** Faults corrected and those not corrected among the 16 types through the use of CCM.

Indicator	CCM v.1	CCM v.2	CCM v.3	CCM v.4
**Ddn**—Discarded data	8	8	6	7
**Ddn**—% Discarded data	50%	50%	37.5%	43.75%
**Ddn**—Corrected data	8	8	10	9
**Ddn**—% Corrected data	50%	50%	62.50%	56.25%

## Data Availability

Data are contained within the article.
